# Increased nocturnal periodic limb movements in rheumatoid arthritis patients meeting questionnaire diagnostic criteria for restless legs syndrome

**DOI:** 10.1186/1471-2474-15-378

**Published:** 2014-11-18

**Authors:** Regina M Taylor-Gjevre, John A Gjevre, Bindu V Nair

**Affiliations:** Division of Rheumatology, Department of Medicine, Royal University Hospital, University of Saskatchewan, Saskatoon, SK Canada S7N 0W8; Division of Respiratory, Critical Care and Sleep Medicine, University of Saskatchewan, Saskatoon, SK Canada S7N 0W8

**Keywords:** Restless legs syndrome, Sleep, Rheumatoid arthritis, Periodic limb movements

## Abstract

**Background:**

Based on questionnaire criteria, the sensorimotor disorder restless legs syndrome (RLS) has been reported to have a higher prevalence in rheumatoid arthritis (RA) patients than in the general population. There has been some speculation that peripheral arthritic symptoms may allow false positive responses to questionnaire criteria. This study evaluates whether RA patients meeting RLS questionnaire criteria also have objective evidence of increased periodic limb movements (PLMs) characteristic of RLS.

**Methods:**

Participants were recruited from RA clinic. Questionnaire data collected at study entry included: pain scores, rheumatoid arthritis disease activity index, Epworth sleepiness scale, Pittsburgh sleep quality index and RLS diagnostic criteria. Each participant was provided a PAM-RL actigraphic monitor, which attached to the ankle. This device was worn for two consecutive nights then returned for data download. Laboratory data including hemoglobin, iron studies, renal function and C-reactive protein levels were collected.

**Results:**

Of the 57 participants, 23 met RLS diagnostic criteria. Those who met RLS criteria demonstrated higher mean frequency of nocturnal PLMs (19.63/hour; SD:21.13) than those who did not meet RLS criteria (11.13/hour; SD:12.10; p = 0.033). There were no significant differences between groups in terms of patient characteristics, disease activity or duration measures. Patients meeting RLS criteria did have poorer sleep quality measures (p <0.001).

**Conclusions:**

RA patients who met RLS diagnostic criteria demonstrated higher frequencies of nocturnal PLMs than RA patients who did not meet criteria for RLS. This finding supports use of the RLS diagnostic criteria in helping to differentiate between RA arthritic symptoms and RLS.

**Electronic supplementary material:**

The online version of this article (doi:10.1186/1471-2474-15-378) contains supplementary material, which is available to authorized users.

## Background

Restless legs syndrome (RLS), also known as Willis Ekbom disease, is a sensorimotor disorder reported at an increased prevalence in people with rheumatoid arthritis (RA). In 1986 Reynolds found 30% of a sample of hospitalized RA patients had RLS [1]. Subsequently in 1994, Salih reported RLS in 25% of RA out-patients [2]. In 2003 the International Restless Legs Syndrome Study Group (IRLSSG) published diagnostic criteria for RLS [3]. Utilizing these RLS criteria in a Canadian RA outpatient cohort, 27.7% of patients were found to meet criteria [4]. RA patients with RLS have been observed to report poorer sleep quality, increased pain, depression, and poorer quality of life scores than those without RLS [5].

The IRLSSG essential diagnostic criteria consist of a four-component questionnaire. All four criteria must be present to make a diagnosis of RLS and include the following: 1. An urge to move the legs, usually accompanied by or caused by uncomfortable and unpleasant sensations in the legs; 2. The urge to move or unpleasant sensations begin or worsen during periods of rest or inactivity such as lying or sitting; 3. The urge to move or unpleasant sensations are partially or totally relieved by movements, such as walking or stretching, at least as long as the activity continues; 4. The urge to move or unpleasant sensations are worse in the evening or night than during the day or only occur in the evening or night [3].

There has been some concern that these essential criteria are vulnerable to inclusion of confounding conditions or ‘mimics’ [6]. Arthritic pain has been suggested as a possible mimic, which may be experienced by patients in a manner allowing them to meet RLS criteria on a questionnaire [6]. Conversely, over 90% of RA patients in a previous study who met RLS criteria felt they could differentiate between these leg symptoms and their arthritic symptoms [4]. However, it may be possible that the presumed higher prevalence of RLS in RA does relate to lower extremity arthritic symptoms being interpreted as RLS features.

In this study we wished to ascertain whether further evidence might be found to distinguish between RLS and ‘mimic’ symptoms in RA patients. As RLS is a sensorimotor disorder, increased periodic limb movements (PLMs) are a characteristic motor finding [7]. We compare the frequency of PLMs as measured by nocturnal actigraphy, between RA patients meeting RLS diagnostic criteria and RA patients not meeting criteria.

## Methods

Participants were recruited from RA outpatient clinics. Of the 109 consecutive RA patients invited to participate, 57 consented and completed the study. The majority of those declining participation expressed disinclination to wear the actigraphic monitor overnight. There were small differences in age (mean age in non-participants was 59.7 years and for participants was 55.1 years) and gender proportion between those who did not (10 males in 52 non-participants) and did (9 males in 57 participants) choose to participate.

Inclusion criteria included: diagnosis of rheumatoid arthritis by a rheumatologist, ability to provide informed consent, and age over 18. Exclusion criteria included: current or recent (within last year) pregnancy and joint replacement within the previous six months. Patients with known diagnoses of Parkinson’s disease, other non-RLS movement disorders, or who utilized dopaminergic therapies were also excluded. RA clinic patients were interviewed to determine interest and eligibility. Upon study entry, an in-person interview to complete the RLS essential diagnostic criteria was conducted. Participants who met all four criteria were considered to have RLS.

Participants also completed a questionnaire instrument, which included the disease specific Rheumatoid arthritis disease activity index (RADAI) [8], a visual analogue scale (VAS) for pain measurement, the Pittsburgh Sleep Quality Index (PSQI) [9], and the Epworth Sleepiness Scale (ESS) [10].

During this study we employed the Physical Activity Monitor- Restless Legs (PAM-RL) actigraphic monitor [Mini Mitter Company Incorporated, A Respironics Incorporated Company] designed to document physical activity associated with movement during sleep. Study participants each received individual training on use of the actigraphic device and the sleep diary. Participants were advised to use the actigraph on one leg for two consecutive nights and record in the sleep diary when they retired for the night, arose after sleep and when during the night they may have gotten out of bed.

The PAM-RL has been validated against polysomnography for use in actigraphic measurements [11]. It is battery powered and contains a three-dimensional piezoelectric sensor assembly that records physical motion. An up/down sensor is also incorporated into the monitor allowing identification of activity in the vertical compared to horizontal position. This small device is attached to the ankle with a Velcro strap. The acceleration signal produced by body motion is sampled at a rate of 40 times/second. Four consecutive samples are then averaged and the result stored in memory at a rate of ten times per second. After the study is completed, the stored data is downloaded using a SmartCable SP serial cable and analyzed utilizing specific PAM-RL software. Recordings taken during the time out of bed overnight were excluded from the data analysis.

Participants were provided with a laboratory requisition for study-related testing upon study entry and receipt of their actigraphic device. Included in the requested studies were complete blood counts (CBC), creatinine, C-reactive protein, and iron studies (serum ferritin, iron saturation, total iron binding capacity, serum iron).

This study was approved by the University of Saskatchewan Biomedical Research Ethics Board, and the Saskatoon Health Region operational research office. Each participant signed a written consent form.

### Statistical analysis

Data was entered and analyzed within SPSS v.19.0. Categorical data was compared between groups using Chi-Square testing, when the cell size was smaller than five, Fishers Exact test was utilized. Distribution of continuous data was compared between groups using Mann Whitney U non-parametric testing. Calculation of sample size utilizing an alpha value of 0.05, beta of 0.20, minimal clinical PLM index difference of 5 and published standard deviation data [7, 11] yielded a sample size requirement estimation of sixteen participants per group [12].

## Results

Of the 57 RA patients who participated in this study, 9 were male (15.8%). The mean age of participants was 55.13 years (SD: 14.9; range 23–87). Of the 57 participants, 23 met the essential diagnostic criteria for restless legs syndrome and 34 did not. These two groups of participants were compared for differences in age, body mass index (BMI), various laboratory parameters, RA disease activity assessments, and in sleep health (Table 1).

**Table 1:** Characteristics of groups dependent on RLS criteria status*

Variable	RLS- (SD) [n = 34]	RLS + (SD) [n = 23]	Significance
Age (years)	54.76 (15.90)	55.65 (13.59)	0.809
Gender [Males: Females]	6:28	3:20	0.468
BMI	27.67 (6.15)	31.25 (8.53)	0.094
VAS pain (cm)	3.34 (2.07)	3.87 (2.24)	0.334
RADAI	2.96 (2.00)	3.66 (2.21)	0.197
PSQI	5.97 (3.47)	9.59 (3.11)	<0.001
ESS	7.42 (4.70)	7.87 (4.02)	0.682
Hemoglobin [g/L]	132.50 (15.38)	136.33 (10.07)	0.418
Ferritin [μg/L]	109.42 (118.67)	103.21 (111.17)	0.818
Iron [μmol/L]	15.44 (7.75)	16.43 (6.62)	0.568
C-reactive protein [mg/L]	6.09 (6.72)	4.02 (6.17)	0.144
Parity in female participants	2.37 (2.42)	1.00 (1.22)	0.071
Duration of RA diagnosis (years)	10.91 (9.38)	14.83 (12.11)	0.225

*Mean values provided for non-categorical data; RLS: restless legs syndrome; SD:standard deviation; BMI: body mass index (kilograms/metre^2^); VAS: visual analogue scale; cm: centimetres; RADAI: rheumatoid arthritis disease activity index; PSQI: Pittsburgh sleep quality index; ESS: Epworth Sleepiness Scale; g: grams; L: litre; μg: microgram; μmol: micromole; mg: milligram.

There were no patients in the RLS group who had elevation in serum creatinine (1 in non-RLS group). Two patients in the non-RLS group had had a previous lower extremity joint replacement (>6 months earlier). One patient in each group was concurrently utilizing a CPAP (continuous positive airway pressure) device for treatment of obstructive sleep apnea (OSA). There was no significant difference between groups for ESS scores. Four patients reported previous diagnoses of RLS. Three of these four met RLS diagnostic criteria on study entry. Of these four patients, one was receiving iron supplements and one amitriptyline for the RLS symptoms. No significant differences in parity were observed between female participants in each group.

There were no significant differences between these two groups in measurements of laboratory parameters, which included: complete blood counts, creatinine, C-reactive protein, and iron studies (serum ferritin, iron saturation, total iron binding capacity, serum iron).

In terms of assessments related to rheumatoid arthritis, there were no statistically significant differences in RADAI scores, or VAS scores for pain between groups.

**Figure 1:**
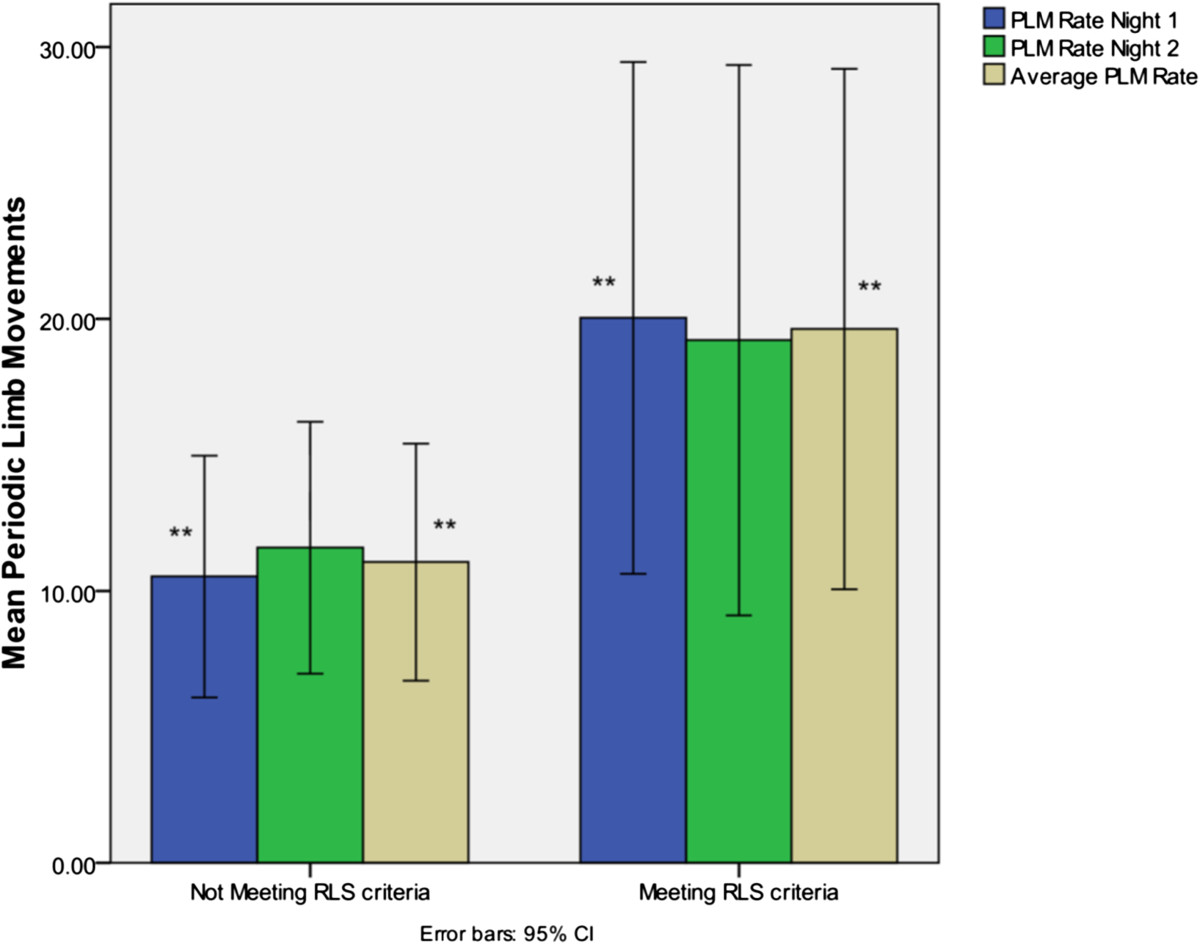
**Periodic limb movements recorded over night 1, night 2 and the averages by patient group.** **indicates significant difference between groups, p < 0.05.

Periodic limb movements/hour and sleep duration for each group are described in Table 2 and Figure 1. Significantly higher mean PLMs were observed in the RA patient group who met RLS diagnostic criteria.

**Table 2:** **Periodic limb movements and sleep duration measures**

Measure	RLS- (SD) [n = 34]	RLS + (SD) [n = 23]	Significance
PLMs/hour Night 1	10.62 (12.35)	20.04 (21.76)	0.015
PLMs/hour Night 2	11.59 (13.05)	19.22 (23.40)	0.088
Average PLMs/hour	11.13 (12.10)	19.63 (22.13)	0.033
Duration of sleep (hours) Night 1	07:47 (01:29)	08:04 (01:12)	0.459
Duration of sleep (hours) Night 2	07:46 (01:32)	07:44 (00:51)	0.588
Average sleep duration (hours)	07:47 (01:27)	07:54 (00:56)	0.890

Mean values provided for non-categorical data; RLS: restless legs syndrome; SD: standard deviation; PLMs: periodic limb movements.

## Discussion

In this study, RA patients who met RLS criteria, demonstrated a higher frequency of nocturnal PLMs than those participants who did not. This finding supports the premise that RLS symptoms can be distinguished from arthritic pain by people with RA. Consequently, these findings are supportive of the previously reported increased prevalence of RLS (25-30%) in RA populations [1, 2, 4].

The proportion of participants meeting RLS criteria in our study is higher than in other questionnaire prevalence studies in RA populations. We expect this likely relates to self-selection bias in patient participation. Those with symptoms suggestive of RLS may have been more likely to agree to participate in this study than those without such symptoms.

The purpose of this study was to compare motor manifestations of RLS in RA patients who did and did not meet RLS criteria. Additionally, this study was not designed or powered to evaluate authoritatively any pathologic or physiologic associations with RLS in this patient population. The physiologic basis for an increased prevalence of RLS in RA patients has not been established. There has been a substantial body of work in other populations identifying iron deficiency states, parity, renal failure and some neurologic diseases to be associated with higher risk for development of RLS [3]. Dopaminergic abnormalities have been hypothesized and dopamine receptor agonists are widely employed as therapeutic agents for RLS [3].

Various medications have been recognized to influence periodic limb movement frequencies in both positive and negative directions. Small numbers of patients in this study were utilizing various psychoactive medications, sedatives, or narcotics. There were no significant differences in use of these medications between study patients meeting RLS criteria and those who did not.

In this study no clear relationship was identified between previously recognized risk associations and RLS. This may be related to the study population size and selection, however it may also reflect under-recognition of disease processes such as subclinical neurologic involvement. There was an observed trend between higher disease duration and activity and RLS. This finding, although not statistically significant may signal a relationship between cumulative exposure to the inflammatory disease or therapies and development of RLS.

This study was conducted using the PAM-RL ambulatory device to detect periodic leg movements. It has been recognized that there is variability in sensitivity between types of actigraphic devices, as well as night-to-night variability in PLM frequency [13, 14]. In order to consider possible ‘first night effect’ and to accommodate potential night-to-night variability in PLM counts the study was conducted over two consecutive nights. Kobayashi in a recent study of actigraphic evaluation in Japanese patients suggests a longer duration of consecutive observation may further increase sensitivity for diagnosis of PLM disorders [13]. Similarly, Gschliesser has reported bilateral actigraphy may provide greater overall result consistency than unilateral studies [14].

A relationship between higher PLM counts or indexes has been observed with sleep restriction and also sleep apnea [3]. We observed comparable sleep durations between groups on study nights although this does not exclude the possibility of previous sleep restriction prior to study nights. Obstructive sleep apnea (OSA) or sleep disordered breathing have been reported as increased in RA patients [5, 15]. Epworth Sleepiness Scale (ESS) scores have been demonstrated to have high predictive capacity for OSA in RA patients [15]. However, in this study population no significant differences in ESS scores were observed between groups.

### Study limitations

#### Study population

The study population may have been influenced by self-selection bias in patient participation. Those patients invited to participate who recognized having symptoms suggestive of RLS may have been more likely to agree to join in the study than those without such symptoms. This potential study population bias may affect extrapolation of findings, particularly in reference to RLS prevalence, to other patient groups.

#### Confounding influences

Although care was taken to exclude potential participants utilizing dopamine agonists or similar therapies used as treatments for RLS, small numbers of participants were utilizing various other medications with the potential to impact PLM assessment by either increasing or decreasing PLMs. These various medications included sedatives, narcotics and psychoactive agents. Although, there were no significant differences in use of these medications between study patients meeting RLS criteria and those who did not, it is possible that use of these or use of other medications (prescription and non-prescription), which may not be currently recognized to affect PLM activity, could have influenced study results in either direction.

#### Measurement sensitivity

As previously mentioned, there has been discussion around the benefits to sensitivity of even longer duration of consecutive night measurements than were used in this study. Bilateral simultaneous actigraphic monitoring has also been suggested to increase sensitivity relative to unilateral limb monitoring, which was employed in this study. Variability in the sensitivity of different types of actigraphic devices has also been recognized [13, 14].

## Conclusions

In this study RA patients meeting RLS essential criteria had significantly increased periodic limb movements compared to RA patients who did not meet these questionnaire criteria. This finding supports consideration of screening RA patients for RLS and also employing the RLS criteria questionnaire for the screening process [5, 15]. Appropriate recognition of RLS and potentially subsequent directed therapies [16, 17] may prove beneficial to quality of life in this patient population.

## Authors’ information

RTG is a Professor of Medicine and Head of Rheumatology at the University of Saskatchewan, JG is an Associate Professor of Medicine in the Division of Respirology, Critical Care, and Sleep Medicine at the University of Saskatchewan, and the Saskatoon Health Region Sleep Laboratory Director, BVN is an Associate Professor and Rheumatologist at the University of Saskatchewan.

## Authors’ original submitted files for images

Below are the links to the authors’ original submitted files for images. Authors’ original file for figure 1

## Abbreviations

BMI: body mass index
CBC: complete blood count
CPAP: continuous positive airway pressure
ESS: Epworth sleepiness scale
IRLSSG: International restless legs study group
OSA: obstructive sleep apnea
PAM-RL: Physical Activity Monitor- Restless Legs
PLMs: periodic limb movements
PSQI: Pittsburgh sleep quality index
RA: rheumatoid arthritis
RADAI: rheumatoid arthritis disease activity index
RLS: restless legs syndrome
SD: standard deviation
SPSS: Statistical package for the social sciences
VAS: visual analogue scale.
