# Supplementary figures and images for: Increased nocturnal periodic limb movements in rheumatoid arthritis patients meeting questionnaire diagnostic criteria for restless legs syndrome
Source: BMC Musculoskelet Disord. 2014 Nov 18;15:378. doi: 10.1186/1471-2474-15-378 (PMC4247724; doi:10.1186/1471-2474-15-378)

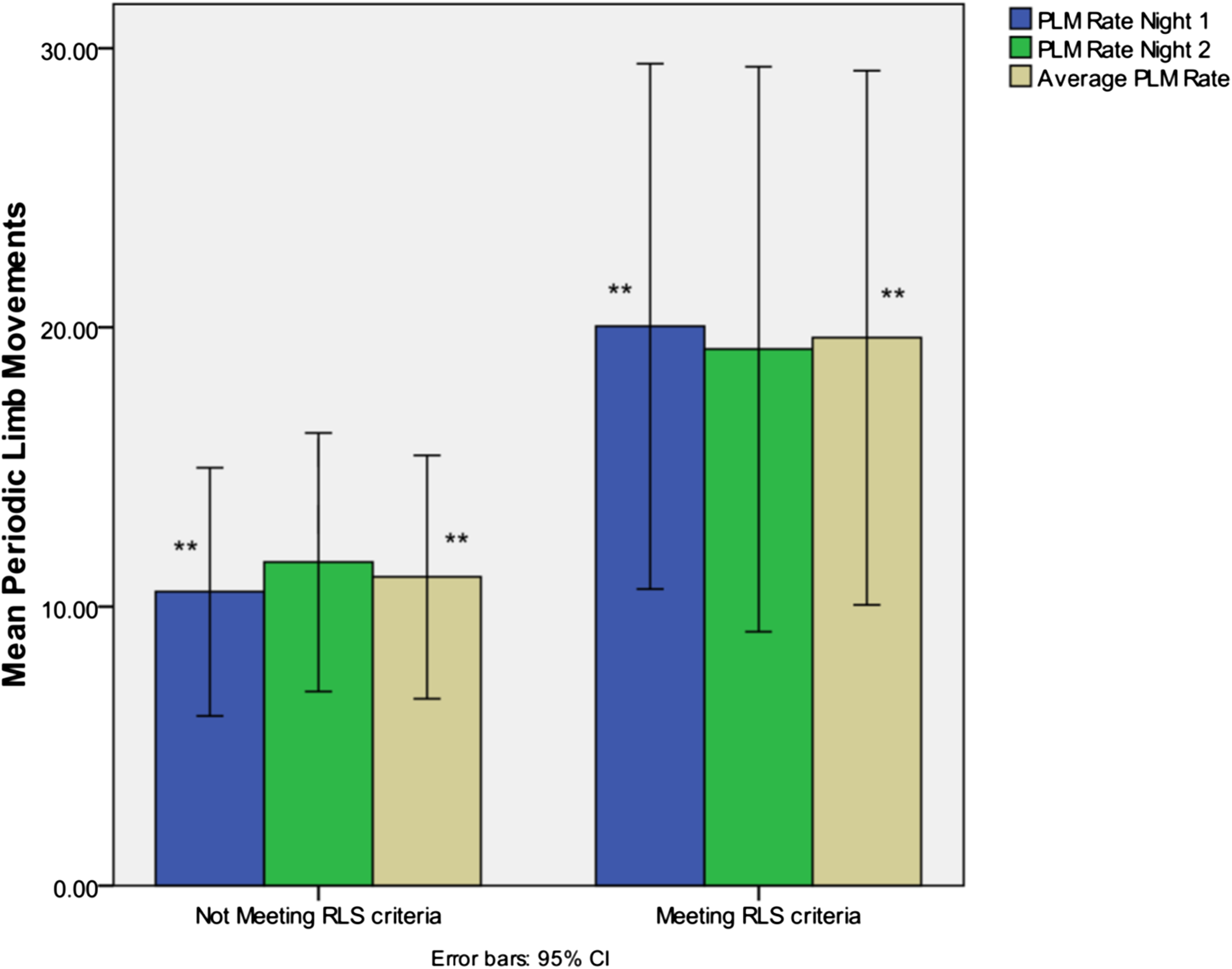

Supplement: Supplementary file 1 — Authors’ original file for figure 1 [file 12891_2014_2324_MOESM1_ESM.tif]
